# Chemical Essays

**Published:** 1870-02

**Authors:** George Watt

**Affiliations:** Professor of Pathology and Therapeutics, late Professor of Chemistry and Metallurgy in the Ohio College of Dental Surgery, etc.


### CHEMICAL ESSAYS.

*Register Papers—A Collection of Chemical Essays in Reference to Dental Surgery.*

BY GEORGE WATT, M.D., D.D.S.,
Professor of Pathology and Therapeutics, late Professor of Chemistry and Metallurgy in the Ohio College of Dental Surgery, etc.
12 mo. pp. 260. Philadelphia: S. S. White, 1868.

This work having been published about two years, it is probable the reading men in our profession have already had the satisfaction of perusing it. It would, therefore, seem almost superfluous to write a review of it; but we have but recently read it, and the reading has prompted a desire to say something in connection therewith. We do not purpose writing a "review," nor yet a mere notice; but if what we may have to say should induce any who have not read the work to do so, we feel assured we shall receive their thanks.

The work is not by any means a systematic treatise on a series of chemical subjects, as some might infer from the abbreviated title—"Chemical Essays,"—but as the title page purports, a collection of papers, hitherto published in the DENTAL REGISTER, relating mostly to chemical and miscellaneous topics, treated from a chemical standpoint. Yet the connection and dependence of most of them is such as to give them in the totality the character of a systematic treatise. They are of practical importance, and the views enunciated may be accepted as orthodox. The profession can not but feel under obligation to Dr. Watt for having presented these papers in book form, elucidating, by the clear light of chemical philosophy, some of the most interesting and intricate questions in Dental surgery.

The first two essays, relating to Oxygen and Hydrogen, deal in " generalities." The style and delineation are more poetic than scientific; these two elements being personified, and followed out, in their varied and multitudinous achievements throughout earth, air and sea. As addresses introductory to the course of college lectures, their object appears to have been to enlist the interest of students by appealing to the imagination, instead of at once entering upon the details of abstruse science, to which they were prefatory.

The next essay treats of " Dental Chemistry," wherein the author presents, in strong light, the importance and uses of chemistry to Dental surgery.

We would like to make some extracts, but will pass on to topics having a more practical bearing :

The two succeeding papers relate to Dental Caries, and the following one is quite an elaborate treatise, of great interest, upon the " Dental Chemistry of the Mouth."

In regard to Dental Caries, we desire to recall attention to one or two points which Dr. Watt enforces so clearly and satisfactorily.

Speaking of caries, now admitted to be the result of chemical action, he says : " When the composition of the teeth is taken into the account, we would infer that the deleterious agents are to be looked for among the acids." But not content with this, as most writers appear to have been, he inquires further—" What acids ? " " Are many acids, or only a few, concerned in its production ? " And again, what are the " various characteristics of those chemical actions which result in what we recognize as Dental Caries ? Do we here find a great variety of appearances ? or is it not well known that the phenomena of Caries are so few, and so circumscribed, that, by common professional consent, but three or four varieties of it are recognized? We find one variety called 'white decay,' and another, that is brownish in color, and a third that is very properly designated as 'black decay.' These differ in other respects as well as color. In the white variety, all the components of the teeth are acted on and disintegrated, as far as the disease extends. In the second variety, the earthy portion of the tooth seems to be removed; while much or all of the animal portion remains, which is conclusive evidence that the chemical agent, whatever it may be, forms soluable compounds with the earthy materials. In the 'black decay,' there is less disintegration of the tooth substance than in either of the other varieties; and it progresses less rapidly than either of them. The physical characteristics of this variety, aside from the chemical, would indicate that the chemical agent principally concerned in its production forms, mainly, insoluble compounds with the constituents of the tooth. Then there is a fourth variety, commonly called 'chemical ebrasion,' in which the entire tooth substance is *removed*, as far as the disease extends. It is evident that the agent producing this, dissolves or forms soluble compounds with both the animal and earthy materials of the tooth."

"The physical characteristics of decay depend much on the texture of the teeth affected; but they are dependent, also, on the nature of the compounds formed by the union of the destroying agent with the constituents of the teeth. The degree of concentration of the chemical agent has also a modifying influence. When much diluted, its action is almost solely in obedience to its strongest affinity. For example, if nitric acid were the agent, when concentrated it would act energetically on the animal as well as the earthy materials of the teeth; but, when much diluted, its action would be almost confined to the latter.

"The chemical characteristics of decay, however, depend almost exclusively on the character of the agent producing it. The truth of this appears evident, when we reflect that bad teeth and good ones are composed of the same chemical substances. Marble and chalk are alike in chemical composition, but not in physical structure ; and, though an acid acts more rapidly on the latter than on the former, yet the result of the action is the same. An acid, too, will act with more energy on a soft, porous tooth than on one of firmer texture ; yet the chemical results are the same. It is safe to conclude, then, that as there are but few *results* in the chemical actions attendant on Dental Caries, there are but few chemical agents immediately concerned in their production."

The writer remarks that it is not to be inferred from the above that but few agents are capable of injuring the teeth by chemical action ; but that many acids used in food or as medicines are capable of doing this—by facilitating or predisposing to the production of Caries—and yet without being the *immediate* cause of the decay. The teeth may be so corroded by them as to expose the dentine and render it liable to the action of the carious agent ; or the enamel may be roughened, either mechanically or chemically, so as to afford a lodgment for organic matter, which, by decomposition, may generate one of the acids immediately concerned in the production of Caries."—pp. 41—44.

In a succeeding paper (" Dental Chemistry of the Mouth "), having detailed the various constituents of the teeth, and shown the peculiar effects of different re-agents upon these constituents, the author designates, and explains the action of, the particular acids productive of the several kinds of decay above mentioned. Nitric acid is the prominent agent in the production of " white decay ; " sulphuric acid of " black decay," and hydrochloric acid of the " brown " variety.

It is exceedingly interesting to trace the peculiar action of these acids, and the manner in which they are generated in contact with the teeth, as so minutely and lucidly described by the author, whose searching philosophy permeates and beautifully illuminates every topic which he touches. We would like to make more copious extracts upon these and other subjects treated, but our limits admonish us to forbear. Besides, it would not do adequate justice to the author, who must be read in order to be duly appreciated; and, in addition to the valuable information contained, his work will teach the attentive reader, by example, how to observe and study the phenomena of nature, and how to apply the principles of chemistry to practical use.

There are several other papers of practical interest—The Topical Treatment of Inflamed Dentine, Alveolar Hemorrhage, Exposed Pulps, Bleaching Teeth, Alloys, The Management of Gold Alloys, Nitrous Oxyd as an Anæsthetic, &c.; also several elaborate papers upon important chemical elements and compounds—Arsenic, Chlorine, Calcium, Nitrate of Silver, &c. Besides, several miscellaneous subjects, all of which will well repay perusal, being pervaded by good sense and genial humor, and enlivened by occasional sallies of inimitable satire.

The work is gotton up in beautiful typographical style, neatly and substantially bound—an ornament to any library. We hail it as a most valuable contribution to our professional literature. B. W.

